# Bayesian Inference of Phylogenetic Distances: Revisiting the Eigenvalue Approach

**DOI:** 10.1007/s11538-024-01403-z

**Published:** 2025-01-23

**Authors:** Matthew J. Penn, Neil Scheidwasser, Christl A. Donnelly, David A. Duchêne, Samir Bhatt

**Affiliations:** 1 Department of Statistics, University of Oxford, Oxford, UK; 2 Section of Epidemiology, University of Copenhagen, Copenhagen, Denmark; 3 Pandemic Sciences Institute, University of Oxford, Oxford, UK; 4 MRC Centre for Global Infectious Disease Analysis, Imperial College London, London, UK

**Keywords:** Bayesian inference, GTR, Genetic distance, Ukaryotes

## Abstract

Using genetic data to infer evolutionary distances between molecular sequence pairs based on a Markov substitution model is a common procedure in phylogenetics, in particular for selecting a good starting tree to improve upon. Many evolutionary patterns can be accurately modelled using substitution models that are available in closed form, including the popular general time reversible model (GTR) for DNA data. For more complex biological phenomena, such as variations in lineage-specific evolutionary rates over time (heterotachy), other approaches such as the GTR with rate variation (GTR$+\Gamma$) are required, but do not admit analytical solutions and do not automatically allow for likelihood calculations crucial for Bayesian analysis. In this paper, we derive a hybrid approach between these two methods, incorporating $\Gamma (\alpha ,\alpha )$-distributed rate variation and heterotachy into a hierarchical Bayesian GTR-style framework. Our approach is differentiable and amenable to both stochastic gradient descent for optimisation and Hamiltonian Markov chain Monte Carlo for Bayesian inference. We show the utility of our approach by studying hypotheses regarding the origins of the eukaryotic cell within the context of a universal tree of life and find evidence for a two-domain theory.

## Introduction

Phylogenetic distance is a fundamental quantity when considering the evolutionary history of a set of taxa. Its definition serves as the cornerstone for constructing phylogenetic trees, which represent the evolutionary relationships between organisms. These trees, represented as bifurcating binary structures (though often unrooted), visually depict the evolutionary distances and genetic relatedness among different species or organisms. In both likelihood and minimum evolution methods, the lengths of the branches - often representing the evolutionary time between bifurcations - and the overall tree topology are ultimately heavily dependent on distance calculations, either directly through the objective function or implicitly in the estimation of the model parameters. Thus, the development of new methods and tools for distance estimation is a crucial area of research for modern phylogenetics (Ferretti et al. 2024).

There are many definitions of phylogenetic distance. The simplest such metric is the Hamming distance which, for a pair of taxa *g* and *h*, simply measures the proportion of sites which take a different value in the two genomes. Although its simplicity is appealing, the Hamming distance ignores the possibility of multiple changes at a given site (“multiple hits") as well as the heterogeneity in the character set $\chi$, which for amino acid or codon sequences is somewhat large.

The solution ubiquitously used to these challenges is to introduce a Markov model for the substitution process, where we suppose that each site evolves according to an independent Continuous-Time reversible Markov Chain (CTMC) with a substitution rate matrix *Q* and a transition matrix *P*(*t*). We suppose that each chain is started at taxon *G* according to the stationary distribution, $\varvec{\xi }$, and that the corresponding site in genome *H* takes the value of the CTMC at time *d*(*g*, *h*), the evolutionary distance between this pair of taxa. From this we can use an objective criteria such as minimum evolution (Day et al. 1986; Pauplin 2000; Desper and Gascuel 2002; Gascuel and Steel 2006; Lefort et al. 2015; Penn et al. 2023) to estimate a phylogenetic tree.

For a range of CTMC models, the genetic distance is available in closed form. For example, under the general time reversible model (GTR), the genetic distances are simply

$$d(g,h) =-\text {tr}(\text {diag}(\varvec{\xi }) \text {log}\left( \text {diag}(\varvec{\xi })^{-1}K^{gh})\right) $$

where $K^{gh}$ is known as the frequency or divergence matrix representing the proportion of changes from the character set $\chi$. When incorporating $\Gamma (\alpha ,\alpha )$-distributed rate variation it is possible to define a formula for the GTR+$\Gamma$ model (Gu and Li 1996; Yang and Kumar 1996) which can be represented in closed form via eigendecomposition, but does not admit an analytical solution. To estimate distances from a GTR+$\Gamma$ model, several approaches have been suggested. It is possible to use empirical rates for the GTR substitution matrix via a simple average (Waddell and Steel 1997; Gatto et al. 2007), but these can be problematic due to negative eigenvalues, which means that adjustments are needed. For the $\Gamma$ parameter, $\alpha$, parsimony can be used (Yang and Kumar 1996). More recently, the parameter $\alpha$, distances and GTR parameters can all be considered free parameters and optimised via gradient descent (e.g. Penn et al. (2023)), but the number of distances grows quadratically with the number of taxa, and optimisation can become intractable quickly. Additionally, for protein residues, the larger character set means there is a risk of overfitting. Alongside the GTR model, another common alternative is the log-det or paralinear model (Lake 1994; Lockhart et al. 1994), which calculates a genetic distance solely from $K^{gh}$ and includes a geometric mean estimator for the frequencies that allows the model to account for heterotachy (that is, differences in evolutionary rate across the tree).

Here we build on the work of Lake (1994), Lockhart et al. (1994), Yang and Kumar (1996), Gu and Li (1996), Waddell and Steel (1997), Gatto et al. (2007), Penn et al. (2023), assimilating their previous results to create a new Bayesian model to estimate genetic distances under a GTR+$\Gamma$ with heterotachy. We first introduce the concept of frequency matrices and rederive the log-det estimator in full. We then rederive an equation for the analog to the log-det estimator under $\Gamma$ variation using a simple spectral approach first introduced by Gu and Li (1996). This results in a closed-form formula for the distance based only on $\alpha$. In previous research (Gu and Li 1996), $\alpha$ is independently estimated and no provisions for heterotachy are made. We present an approach that can model GTR+$\Gamma$ with heterotachy and utilises the standard Markov likelihood for distance models to learn $\alpha$ and the GTR rates from the data. We then introduce a Bayesian hierarchical model that puts a prior distribution on GTR rate parameters and $\alpha$ but uses an estimator for $\varvec{\xi }$. Through implementing SVD decompositon, we mitigate the numerical issues from using GTR rates computed directly from the data (Gatto et al. 2007), ensuring that we always get a valid eigendecomposition. Moreover, allowing $\varvec{\xi }$ to vary from pair to pair, our model also accounts for heterotachy. Thus, our model is a hybrid between the log-det-style models and common estimation approaches for the standard GTR+$\Gamma$ from Gu and Li (1996).

## Summary and Results

Broadly, we aim to estimate the evolutionary distance between each pair of taxa given a genetic sequence alignment. We do this by using a large-sites approximation, where we essentially assume that the observed transitions are (in proportional terms) equal to their expected values according to our site mutation CTMC. This approximation enables us to derive an equation for the distance between each pair of taxa, uniquely defining them in terms of the other model parameters.

Thus, when we construct a likelihood to sample our model parameters, we do not need to separately sample our distances. This is a substantial reduction in the number of free parameters, particularly when the number of taxa is large. We detail our implementation and results below, and our full derivations in the subsequent “Methods” section.

### Bayesian Distance Estimation

Given our set of genetic sequences $G^1,...,G^n$, each of length $N>>1$, we first calculate pairwise frequency matrices

1 $$\begin{aligned} K^{ab}_{ij} = \frac{1}{N}\sum _{k} \mathbb {I}\left\{ G^a_k = i\right\} \mathbb {I}\left\{ G^b_k = j\right\} \end{aligned}$$

and simple frequency vectors

2 $$\begin{aligned} F^a_i = \frac{1}{N}\sum _k \mathbb {I}\left\{ G^a_k = i\right\} \end{aligned}$$

for each pair of taxa *a* and *b*. Note $\varvec{F}^a$ and $\varvec{F}^b$ are simply the row and column sums of $K^{ab}$.

Using these inputs, which need only be calculated once (and in a manner that can be parallelised), we can then define a hierarchical Bayesian model. Our framework is not specific to any particular choices on the prior distributions. However, in the examples in this paper, we construct the Q-matrix *Q* (i.e. the instantaneous transition matrix) from a rate matrix *S*, and the stationary distribution $\varvec{\xi }$ via the operations

$$\begin{aligned} Q^1&= S\text {diag}(\xi )\\ Q^2&= Q^1- \text {diag}\left( Q^1\varvec{1}\right) \\ Q&= \frac{Q^2}{\sum _iQ^2_{ii}\xi _i} \end{aligned}$$

where here superscipts denote steps in the process of constructing *Q*.

We also use $\Gamma (\alpha ,\alpha )$-distributed rate variation across sites. These parameters are assigned prior probabilities as follows

$$\begin{aligned} S&\sim \text {Normal}^+(\text {LG},1)\\ \alpha&\sim \text {HalfNormal}(1). \end{aligned}$$

We choose the LG model (Le and Gascuel 2008) as the mean in our Normal distribution prior over a GTR (Tavaré 1986) rate matrix. Note that while GTR was initially developed for nucleotides, one can apply the same principles to protein models - allowing a general Q- matrix *Q* and stationary distribution $\varvec{\xi }$ and simply imposing the reversibility conditions $Q_{ij}\xi _i = Q_{ji}\xi _j$. Effectively, this means that only the lower-triangular portion of the rate matrix *Q* contains “free” parameters. Nevertheless, this full GTR model has a free parameter for every pair of taxa and therefore quickly becomes unfeasible for large trees.

To make the GTR model symmetric and eigendecomposable we make the substitution matrix *Q* from the parameter rates *S* (which are shared across all pairs), and a pair-specific stationary distribution estimate $\varvec{\xi }^{ab}$ given by

3 $$\begin{aligned} \varvec{\xi }^{ab}_i = \sqrt{F^a_iF^b_i}. \end{aligned}$$

While phenomena such as heterotachy do not affect the stationary distribution, more complex variation in evolutionary dynamics across the tree could do so. Using these pair-specific estimators of $\varvec{\xi }$ means that our model is therefore more robust to these changes.

An eigendecomposition on a symmetric form of *Q* (see Lemma 1) therefore yields a unique substitution matrix for each taxon pair, i.e. $Q^{ab} = U^{ab} \Lambda ^{ab} (U^{ab})^{-1}$. From this decomposition, we can compute the transition matrix of a path length $t_{ab}$ between taxa *a* and *b* under Gamma rate variation among sites as

4 $$\begin{aligned} P(t_{ab}) = U^{ab} \left( 1 - \frac{t_{ab}\Lambda ^{ab}}{\alpha }\right) ^{-\alpha } (U^{ab})^{-1}. \end{aligned}$$

This decomposition needs to be performed for all pairs (*a*, *b*), and is the main bottleneck in our approach. Next we compute the matrix

5 $$\begin{aligned} \bar{L}^{ab} = \frac{1}{2}\left[ \text {diag}(\varvec{F}^a)^{\frac{1}{2}}K^{ab} \text {diag}(\varvec{F}^a)^{-\frac{3}{2}} + \text {diag}(\varvec{F}^b)^{\frac{1}{2}}K^{ab} \text {diag}(\varvec{F}^b)^{-\frac{3}{2}} \right] \end{aligned}$$

This allows us to define the singular value decomposition $U^{ab}\Sigma ^{ab} V^{ab}=\bar{L}$, giving the distance as

6 $$\begin{aligned} D_{ab} = \frac{1}{2 \text {tr}(\Lambda ^{ab})}\sum _{i=1}^n \bigg [\alpha \left( 1-(\Sigma ^{ab}_{ii})^{-\frac{1}{\alpha }}\right) \bigg ]. \end{aligned}$$

Note, these singular values are computed only once. Finally, our log-likelihood is

7 $$\begin{aligned} \mathcal {L}(K | \alpha ,S) = \sum _{a,b \in \Omega } K^{ab} \cdot \log \left( P(D_{ab})\right) \end{aligned}$$

where here, $\cdot$ is used to denote a dot product between these two matrices, and the logarithm is taken component-wise.

Using this likelihood with our prior probabilities we can define a posterior distribution $p(\alpha ,S | K)$ which is differentiable and can therefore use state-of-the-art Markov chain Monte Carlo (MCMC) samplers such as No-U Turn Sampler Hamiltonian Monte Carlo (Hoffman and Gelman 2014). For each distance matrix in this posterior sample, either a unique “best-fit” tree can be estimated (Penn et al. 2023) through the balanced minimum evolution framework. While this posterior distribution will not reflect the entire variability in the space of trees, as we do not consider the uncertainty in the optimal tree given a distance matrix, it is an effective way of including uncertainty in the distance information, which is of particular importance for difficult-to-estimate long branch lengths (Mossel et al. 2011).

Note that, throughout the derivation of the model, we use the assumption that our model is time-reversible. This is necessary both to avoid overfitting (as it reduces the number of parameters) and to reduce the computational cost. However, the use of our pair-specific stationary distributions (3) helps our inter-taxa distances to be as robust when the reversibility assumption does not hold. This helps to make our model as flexible as possible, while remaining within the practical constraints of the GTR framework.

### A Simulated Example

To begin, we consider an example based on simulated data. We sample trees uniformly at random, with 50 taxa and with uniformly distributed branch lengths. We rescaled the mean root-to-tip distances to 0.3, which is a sensible value for real data (Klopfstein et al. 2017). We them simulate amino acid sequences down this tree with 10,000 sites under an LG model, and a 4-category discrete Gamma distribution with shape sampled uniformly between 0.1 and 5. We optimise the log-likelihood in Eq. (7) using L-BFGS-B optimisation (Varelas and Dahito 2019). In addition, we also treat frequencies and rates has free parameters and optimise a full GTR model.

**Fig. 1:**
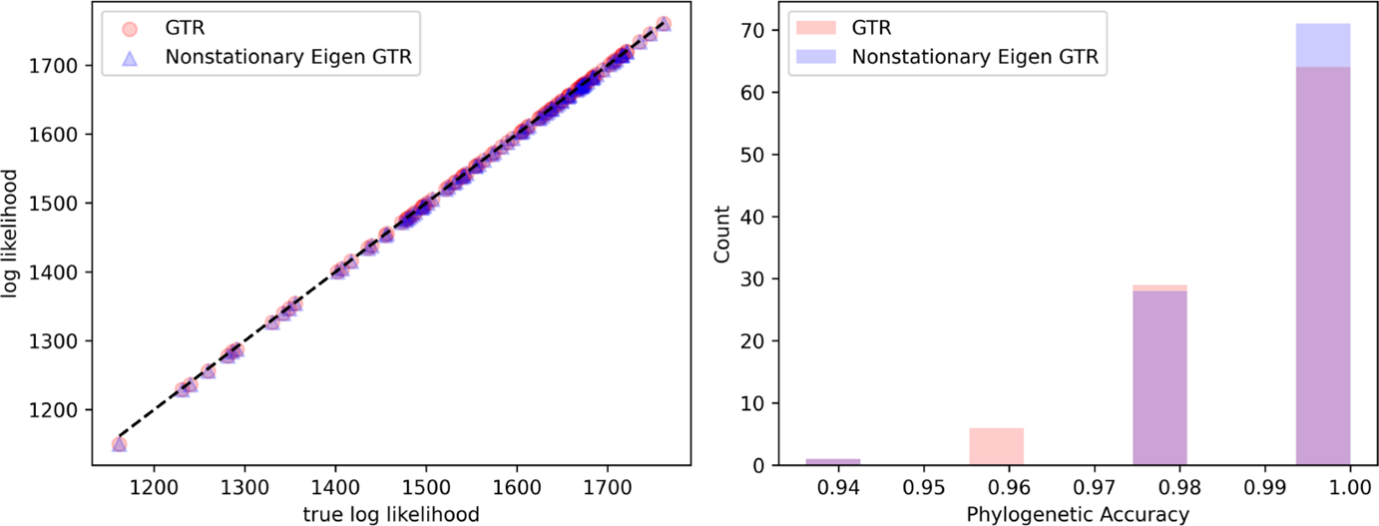
(left) Comparison of our proposed non-stationary Eigen GTR and a full GTR trained with free parameters for every taxa pair with the true log-likelihood for the actual data. (right) Phylogenetic accuracy measured as one minus the RF distance to the true tree from both the non-stationary Eigen GTR and full GTR (Color Figure Online)

Given our optimised inter-taxa distances, we then use FastME (Lefort et al. 2015) to recover an approximate tree. Figure 1 shows that our approach and the GTR model results in virtually the same likelihood as for the true data and the resultant trees are close to the ground truth for both models.

### Comparison to Contemporary Methods

The amino acid example above would be extremely slow to optimise using maximum likelihood-type approaches such as RAxML (Stamatakis 2014) due to the far larger number of parameters. The key strength of our approach is its computational efficiency, with the inter-taxa distances being defined by the rates *S* and gamma shape $\alpha$ (a full discussion of the complexity of our approach can be found in Appendix B). Thus, while we expect that maximum likelihood approaches will out-perform the eigenvalue methods given unlimited computation time, the eigenvalue methods provide a competitive alternative that can be applied to problems with substantially higher number of taxa, and possible site values.

To illustrate the utility of our new approach, we perform the same simulation as above, but instead on nucleotide data using a Jukes-Cantor model of evolution. We then compare both our Eigen and Non-stationary Eigen approach to maximum likelihood (the state-of-the-art) as well as the alternative approach introduced in Penn et al. (2023). We note other distance based approaches exist such as those by Waddell and Steel (1997), but there were no standard implementations of these, and the inclusion of Gamma variation is not trivial to implement.

To provide a fairer comparison, we use the much faster L-BFGS-B maximiser to that chosen in Penn et al. (2023). This faster optimiser leads to poor performance, with a number of the leaf-to-leaf distances being wrong by a substantial margin. This highlights the fact that treating each leaf-to-leaf distance as a free parameter requires computationally intensive optimisation routines, alongside the obvious limitation of the number of parameters scaling quadratically with the number of taxa.

**Fig. 2:**
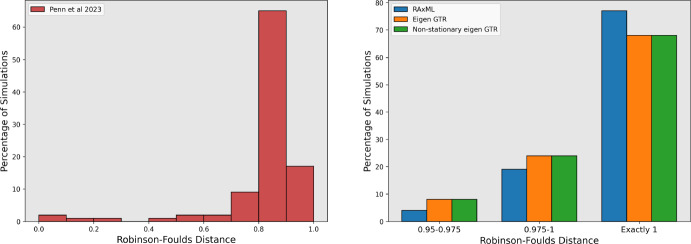
A comparison of the performance on simulated nucleotide dataset by the methods introduced in this paper, alongside RAxML and the inter-taxa estimation method used in Penn et al. (2023). We display histograms of the normalised Robinson-Foulds distance between either the tree created. Note the difference in x axis scale between the two graphs (Color Figure Online)

Again, we use FastME to approximate the true tree once distances have been calculated (with the exception of RaXML, which uses maximum likelihood to find the optimal tree). We show the phylogenetic accuracy (1 minus the Robinsons Foulds distance) in Fig. 2 below. As mentioned above, we observe poor performance from Penn et al. (2023). Excluding this method, in the right-hand panel, we see that, as expected, RAxML performs the best in estimating the correct topology. However, both of our new methods also perform excellently, providing confidence that they would yield good results if applied to a much larger dataset.

Comparing the runtimes of the different algorithms is also notable. RAxML required approximately 6.7 s per run (that is, to generate the optimised tree and protein substitution model parameters from a sequence alignment, using option PROTGTR), considerably higher than the 857 ms that each of the eigenvalue methods required, notwithstanding the fact that RAxML has been far more carefully optimised than the code that runs our new methods. This provides clear evidence for the utility and, potentially, the necessity, of our new methods when analysing large phylogenetic datasets. Furthermore, our approach, as all distance based methods, scales in the number of taxa and not in the number of sites as maximum likelihood methods - enabling genome wide analysis.

### Applications to a Protein-Based Dataset

**Fig. 3:**
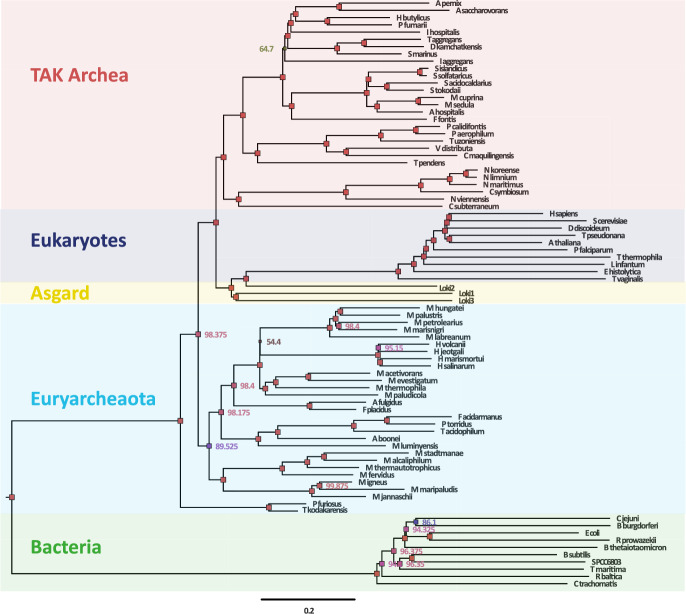
Reanalysis of Williams et al. (2019) studying hypotheses regarding the origins of the eukaryotic cell within the context of a universal tree of life. Using our GTR+$\Gamma$ with hereotachy and prior shrinkage, we find support for two domains of life (Color Figure Online)

**Fig. 4:**
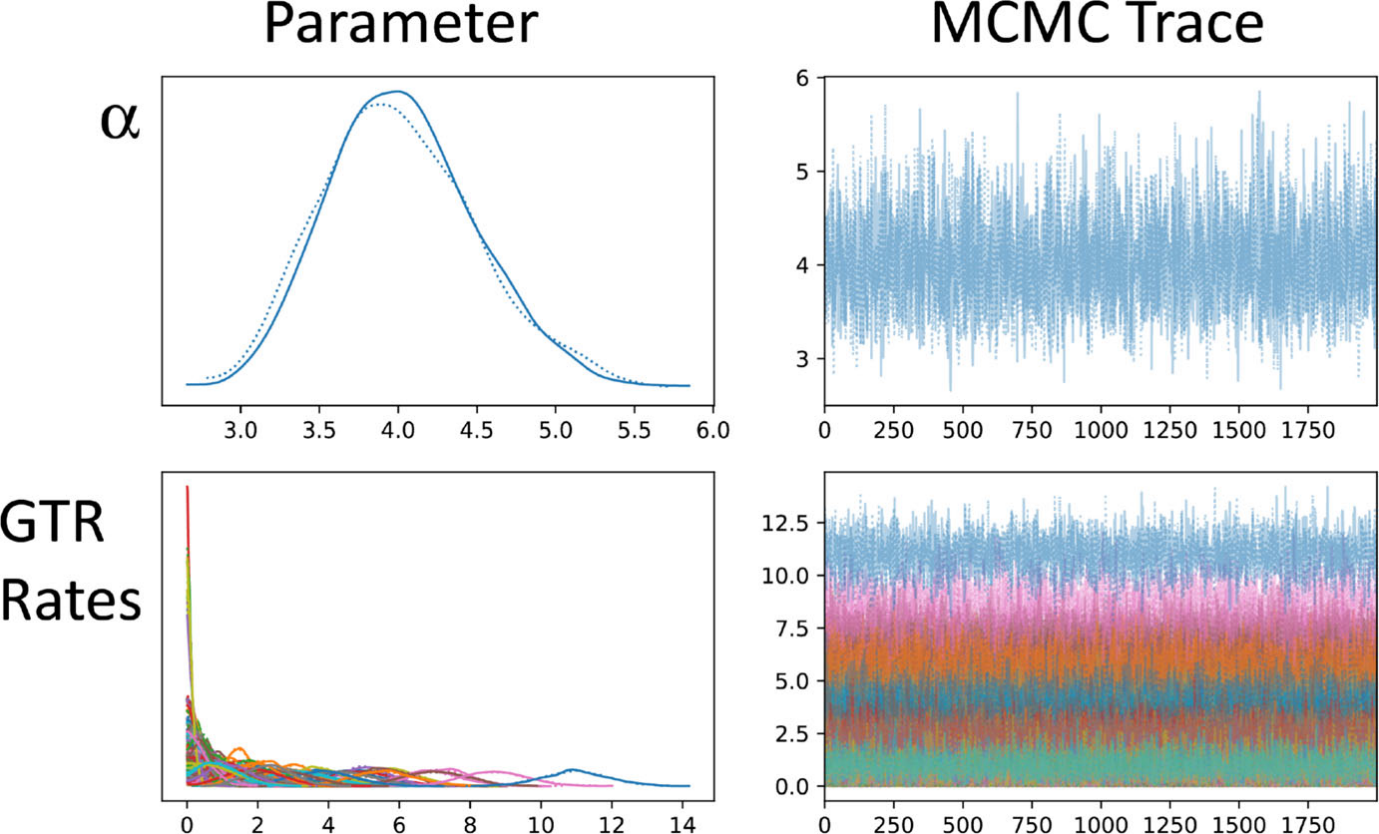
MCMC trace file showing excellent convergence. Right column plots show the trace files, while left column plots the parameters. Dashed and solid lines represent two independent chains (Color Figure Online)

Secondly, we apply our approach to the dataset and problem introduced by Williams et al. (2019) studying hypotheses regarding the origins of the eukaryotic cell within the context of a universal tree of life. Using their dataset comprised of 35 core genes ($\sim$ 7000 protein residues) across 83 taxa, we can apply our Bayesian eigenvalue approach. In their original analysis, Williams et al. (2019) find a GTR + $\Gamma$ + F model yields strong support for a three domain tree of life, but discuss idiosyncrasies in the data that necessitate more sophisticated models of molecular evolution, and under these find stronger statistical support for a two-domain tree of life. Analysing the same data as Williams et al. (2019) we first note that a basic log-det distance estimator (Lake 1994) results in several negative branches between taxa and therefore is invalid. Using an LG model and recovering a bootstrapped distance-based tree via balanced minimum evolution in FastME (Lefort et al. 2015), we find that this tree strongly supports a three-domain tree of life. However, applying our new Bayesian distance-based algorithm, using a GTR model but with an LG prior, results in a two-domain tree (see Fig. 3) with 100% posterior support (though it should be noted that only uncertainty in the distances has been considered in this posterior). While differences exist between our tree and the tree from Williams et al. (2019), and in general we do not expect distance-based approaches to match those based on Felsenstein’s likelihood, our approach shows that distance-based approaches can include important molecular evolution processes such as rate variation and heterotachy. Importantly, due to the ability to utilise state-of-the-art MCMC approaches, our posterior which included 2000 samples, and two chains shows excellent convergence with no pathologies (see Fig. 4).

## Methods

In this section, we provide full derivations of the method in the previous section, alongside examples of how our framework could be applied to a range of other models. As stated in the introduction, this methods section amalgamates the work of numerous previous papers, with the key novelty being the application of log-det-style methods to more complex phylogenetic models.

Note that throughout this methods section, we use the term Q-matrix to refer to the instantaneous substitution matrix of a CTMC.

### Setup

We suppose that the “baseline” mutation CTMC has Q-matrix *Q* and transition matrix *P*(*t*). When we allow rate variation, we will suppose that the true CTMC at a given site has Q-matrix $\tau Q$ for some

$$\begin{aligned} \tau \sim \Gamma (\alpha ,\alpha ) \end{aligned}$$

where the value of $\tau$ is independent of all other sites. Thus, $\tau$ represents the rate at each site in comparison to the mean evolutionary rate. Under this model, the transition matrix becomes $\Pi (t) = P(t/\tau )$.

Note that the stationary distribution, defined to be $\varvec{\xi }$, of our CTMC does not depend on the value of $\tau$ (as if $\varvec{\xi }^T Q = 0$ then $\varvec{\xi }^T (Q\tau ) = 0$ also).

Throughout the majority of this paper, we will work in the large-sites limit. All limiting results for random variables will be under almost-sure convergence.

#### Frequency Matrices

We consider a set of *n* taxa and suppose that each taxon has a (random) genomic sequence $G^1,...,G^n$ with *N* total sites for each of them (though the same methods could easily be applied to, for example, proteomic data).

For each pair of taxa (*a*, *b*), we can define a frequency matrix $K^{ab}$ such that

$$\begin{aligned} K^{ab}_{ij} = \frac{1}{N}\sum _{k} \mathbb {I}\left\{ G^a_k = i\right\} \mathbb {I}\left\{ G^b_k = j\right\} \end{aligned}$$

Thus, $K^{ab}_{ij}$ gives the proportion of sites which take value *i* in taxon *a* and take value *j* in taxon *b*. As an example, the sequences

$$\begin{aligned} G^a = (1,1,2,3) \quad \text {and} \quad G^b = (1,2,4,1) \end{aligned}$$

would lead to

$$\begin{aligned} K^{ab} = \frac{1}{4}\begin{pmatrix} 1& 1& 0& 0\\ 0& 0& 0& 1\\ 1& 0& 0& 0\\ 0& 0& 0& 0 \end{pmatrix} \end{aligned}$$

#### Taking the Large-Sites Limit

Consider the large-sites limit $N\rightarrow \infty$. As

$$\begin{aligned} K^{ab}_{ij} =\frac{1}{N}\sum _{k} \mathbb {I}\left\{ G^a_k = i\right\} \mathbb {I}\left\{ G^b_k = j\right\} \end{aligned}$$

is a sum of independent random variables (assuming that each site evolves independently) we can use the Strong Law of Large Numbers (SLLN) (Gut 2006) to show that

$$\begin{aligned} \mathcal {K}^{ab}_{ij}:= \lim _{N\rightarrow \infty }\left( K^{ab}_{ij}\right) = \mathbb {P}\bigg ( \mathbb {I}\left\{ G^a_k = i\right\} \mathbb {I}\left\{ G^b_k = j\right\} = 1\bigg ) \end{aligned}$$

which, if $X_t$ is a copy of the (general, scaled) mutation CTMC at time *t* and $D_{ab}$ is the distance between taxa *a* and *b*, can be rewritten as

8 $$\begin{aligned} \mathcal {K}^{ab}_{ij} = \mathbb {P}\left( X_0=i,X_{D_{ab}} = j\right) = \xi _i\Pi _{ij}\left( D_{ab}\right) \end{aligned}$$

Note that, even if sites do not evolve independently, extensions of the SLLN exist for various classes of weakly dependent random variables, some of which may be appropriate for phylogenetic modelling (Philipp and Stout 1975) (if, for example, dependence between sites decayed quickly with distance within the genome).

For the remainder of this paper, we will assume that *K* approximates $\mathcal {K}$ well, and will therefore take it to (at least approximately) satisfy (8).

### The Log-Determinant

#### Derivation

Firstly, we consider the case with no rate variation, so that $\Pi = P$. Equation (8) can then be rewritten succinctly in matrix form as

9 $$\begin{aligned} K^{ab} = P(D_{ab}) \text {diag}(\varvec{\xi }) \end{aligned}$$

where for a vector $\varvec{v}$, $\text {diag}(\varvec{v})$ refers to a diagonal matrix $\Delta$ with non-zero entries $\Delta _{ii} = v_i$.

We seek to use this equation to find $D_{ab}$ without relying on the parameters of the CTMC. Note that, by the forward equations for a CTMC,

$$\begin{aligned} \frac{d P(t)}{dt} = QP(t) \quad \text {and} \quad P(0) = I \end{aligned}$$

which has a solution

$$\begin{aligned} P(t) = \exp (Qt)P(0) = \exp (Qt)I \end{aligned}$$

Recall that for a matrix *A* with eigenvalues $\lambda _i$

$$\begin{aligned} \text {det}(e^{A}) = \prod _i e^{\lambda _i} = e^{\text {tr}(A)} \end{aligned}$$

Therefore,

$$\begin{aligned} \text {det}(P(t)) = e^{\text {tr}(Qt)} = e^{\text {tr}(Q) t} \end{aligned}$$

Now, using (9)

$$\begin{aligned} \text {det}(K^{ab}) = e^{\text {tr}(Q) D_{ab}} \text {det}(\text {diag}(\varvec{\xi })) \end{aligned}$$

which rearranges to

10 $$\begin{aligned} D_{ab} = \frac{\log ( \text {det}(K^{ab}) ) - \log (\text {det}(\text {diag}(\varvec{\xi }))}{\text {tr} (Q)} \end{aligned}$$

In this model, there is a degree of freedom in the parameters as one can rescale time (which results in multiplying *D* by some $\hat{t}$ and *Q* by $1/\hat{t}$). In (10), the term $\text {tr}(Q)$ can be treated as this scaling parameter, and therefore without loss of generality, we can set it equal to a convenient value. In this paper, we shall choose $\text {tr}(Q) = -C$, where *C* is the number of possible values that a given site could take (noting that, as it comes from a Q-matrix, $\text {tr}(Q)$ must be negative). Thus, we have

$$\begin{aligned} D_{ab}= \frac{1}{C}\bigg [\log \left( \text {det}(\text {diag}(\varvec{\xi })\right) -\log \left( \text {det}(K^{ab}) \right) \bigg ] \end{aligned}$$

#### Estimating $\varvec{\xi }$

To complete our independence from the parameters of the CTMC, we must find a way to estimate $\log \left( \text {det}(\text {diag}(\varvec{\xi })\right) = \sum _{i=1}^C\log (\xi _i)$. We do this by using the frequency of sites $\varvec{F}^a$ and $\varvec{F}^b$ at each of the taxa where, for example

$$\begin{aligned} F^a_i = \frac{1}{N}\sum _k \mathbb {I}\left\{ G^a_k = i\right\} \end{aligned}$$

There are a plethora of possible consistent estimators that one could use - perhaps the most natural being the arithmetic mean of frequencies between the pairs *a*, *b*

$$\begin{aligned} \varvec{\xi } \approx \frac{1}{2}\left( \varvec{F}^a + \varvec{F}^b\right) \end{aligned}$$

As we will show later in this section, in the log-det formula, we need to estimate $\log (\varvec{\xi })$. Thus, it is natural to instead use

$$\begin{aligned} \log (\varvec{\xi }) \approx \frac{1}{2}\bigg (\log (\varvec{F}^a) + \log (\varvec{F}^b)\bigg ) \end{aligned}$$

Using this estimator is equivalent to taking the average of the distance estimates resulting from $\varvec{\xi } \approx \varvec{F}^a$ and $\varvec{\xi } \approx \varvec{F}^b$.

In effect, this relaxes our reversibility assumption, no longer assuming that the distribution of the sites is the same at taxon *a* and taxon *b*. Indeed, one can note that throughout the derivation, we only used the fact that $\varvec{\xi }$ was the frequency distribution at taxon *a* (although we have tacitly assumed that *Q* is diagonalisable, which is not guaranteed for non-reversible chains). When comparing distant taxa, whose distribution of sites may well not be the same, this can increase the accuracy of distance approximations (Baake 1998), as we are not imposing a constant stationary distribution across the entire tree.

#### The Classical Log-Det Metric

Thus, we recover the classical log-det distance, introduced in (Lake 1994; Lockhart et al. 1994)

$$\begin{aligned} D_{ab}:= \frac{1}{2C}\log \bigg (\text {det}\bigg [\text {diag}(\varvec{F}^a)\bigg ]\text {det}\bigg [\text {diag}(\varvec{F}^b)\bigg ]\bigg )- \frac{1}{C}\log \left( \text {det}(K^{ab})\right) \end{aligned}$$

Note that from the almost-sure convergence of $K^{ab}$ to $\mathcal {K}^{ab}$, the almost-sure convergence of our estimator for $\varvec{\xi }$, and the continuity of Eq. (10), the log-det distance must converge to the true distance in the $N\rightarrow \infty$ limit.

In Appendix C, we explore the metric properties of the log-determinant. We show that for finite *N*, it may not satisfy the triangle inequality, but in the $N \rightarrow \infty$ limit, it is a true metric.

### Rate Variation Across Sites

As previously discussed, it is common to incorporate rate variation across sites. Recall that

$$\begin{aligned} P(t) = \exp (Qt) \end{aligned}$$

Incorporating rate variation involves integrating over a probability distribution function $p(\tau )$ for the scale $\tau$

$$\begin{aligned} P(t) = \int _0^{\infty }\exp (Qt\tau )p(\tau ) d\tau \end{aligned}$$

If $\varvec{v}$ is an eigenvector of *Q* with eigenvalue $\lambda$, then note that

$$\begin{aligned} P(t)\varvec{v} = \int _0^{\infty }e^{\lambda t\tau } p(\tau ) d\tau \end{aligned}$$

and hence

11 $$\begin{aligned} \text {det}(K^{ab}) = \prod _{i}\bigg (\int _0^{\infty }e^{\lambda D^{ab} \tau } p(\tau ) d\tau \bigg )\text {det}\left( \text {diag}(\varvec{\xi })\right) \end{aligned}$$

Unlike in the GTR case, it is impossible to infer the distance $D_{ab}$ without knowing the parameters $\lambda _i$. Treating these as free variables in an optimisation scheme does work reasonably well in practice (that is, attempting to find not only the distances that maximise the likelihood, but the values of the $\lambda _i$ as well). However, inverting these equations carries reasonable computational cost, while accurately finding the $\lambda _i$ requires many inter-taxa pairs to be used simultaneously.

### Gamma-Distributed Rate Variation

We can simplify (11) by supposing that $p(\tau )$ is the pdf of a Gamma-distributed random variable with shape and scale $\alpha$. One could, in principle, simplify this further by using a discrete approximation to the Gamma distribution, but this has been shown to give biased results (Ferretti et al. 2024).

From Lemma 1, we know that *Q* is diagonalisable and so can diagonalise it through the the matrix of eigenvectors *U*. Then,

$$\begin{aligned} P (t) = \int _0^{\infty }\exp (Q t \tau ) p(\tau ) d\tau&= \int _0^\infty U\text {diag}\bigg (\exp \left( \varvec{\lambda } t \tau \right) \bigg )U^{-1} p(\tau )d\tau \\&= U \text {diag}\bigg (\left( 1-\frac{\varvec{\lambda } t}{\alpha }\right) ^{-\alpha } \bigg )U^{-1} \end{aligned}$$

Hence,

12 $$\begin{aligned} \text {det}(K^{ab}) = \prod _{i\in \chi } \left( 1-\frac{\lambda _i D_{ab}}{\alpha }\right) ^{-\alpha } \text {det}(\text {diag}(\varvec{\xi })) \end{aligned}$$

While still an implicit equation, it is computationally easier to solve for the distances in this case as there is no need for the integral.

### An Alternative Approach to Estimating Distances

We can improve on the result in (12) through an eigenvalue approach. Note that, as in, for example (Gatto et al. 2007),

$$\begin{aligned} M^{ab} := K^{ab}\text {diag}(\varvec{\xi })^{-1} = P(t) \end{aligned}$$

where we can treat *M* as a known matrix by estimating $\varvec{\xi }$ from the frequency vectors $\varvec{F}$. We assume that *M* can be diagonalised and has eigenvalues $\mu _1,...,\mu _N$. Now, equating these to the eigenvalues of *P*(*t*), we get a system of equations

$$\begin{aligned} \mu _i = \bigg (1 - \frac{\lambda _i D_{ab}}{\alpha }\bigg )^{-\alpha } \end{aligned}$$

and hence

$$\begin{aligned} \lambda _i D_{ab}= \alpha \left( 1 - \mu _i^{-\frac{1}{\alpha }}\right) \end{aligned}$$

Summing these equations over *i* results in

$$\begin{aligned} \text {tr}(Q)D_{ab} = \sum _{i=1}^n \alpha \left( 1-\mu _i^{-\frac{1}{\alpha }}\right) \end{aligned}$$

Again setting the trace of *Q* to be equal to *C*, we get

$$\begin{aligned} D_{ab} = \frac{1}{C}\sum _{i=1}^n \alpha \left( 1-\mu _i^{-\frac{1}{\alpha }}\right) \end{aligned}$$

which provides an estimate for the distance that has parametric dependence only on $\alpha$. Note that in the $\alpha \rightarrow \infty$ limit, we recover

$$\begin{aligned} D_{ab} = \sum _{i=1}^n \alpha \left( 1-e^{-\frac{\log (\mu _i)}{\alpha }}\right) \sim \sum _{i=1}^n \log (\mu _i) = \text {log-det}(M) \end{aligned}$$

which is the standard log-det metric. This equation was first derived by Gu and Li (1996) and has not been used widely. Our novel contribution is to use the idea of Gu and Li (1996) to create a non-stationary estimator which can be used within a Bayesian model to allow shrinkage.

### Practical Eigenvalue Calculation

Note that when computing the eigenvalues $\mu _i$, $M^{ab}$ is non-symmetric and can have high condition numbers. To increase stability, we find a symmetric matrix with the same eigenvalues. This can be done by noting that (as shown in Lemma 1), from reversibility, the matrix

$$\begin{aligned} R:= \text {diag}(\varvec{\xi })^{\frac{1}{2}}Q \text {diag}(\varvec{\xi })^{-\frac{1}{2}} \end{aligned}$$

is symmetric. Thus,

$$\begin{aligned} \text {exp}(R) = \text {diag}(\varvec{\xi })^{\frac{1}{2}}P \text {diag}(\varvec{\xi })^{-\frac{1}{2}} \end{aligned}$$

is symmetric, and also similar to *P*, meaning it has the same eigenvalues. We therefore also know that

$$\begin{aligned} L := \text {diag}(\varvec{\xi })^{\frac{1}{2}}K^{ab} \text {diag}(\varvec{\xi })^{-\frac{3}{2}} = \text {diag}(\varvec{\xi })^{\frac{1}{2}}P \text {diag}(\varvec{\xi })^{-\frac{1}{2}} \end{aligned}$$

is (at least approximately) symmetric. Thus, we can use this to calculate the values $\mu _i$ in a more numerically stable fashion than directly using *M*.

On smaller datasets, even the matrix *L* can cause numerical problems due to being poorly conditioned. We have found that it is better to use the singular values of *L* (which, if it were symmetric, would coincide with the eigenvalues) to approximate $\mu _i$ in the most stable fashion.

### Estimating $\varvec{\xi }$

Again, these distance estimates rely on the value of $\varvec{\xi }$ and so this must be approximated. As before, we take the average of the distances given by using $\varvec{\xi } \approx \varvec{F}^a$ and $\varvec{\xi } \approx \varvec{F}^b$. Thus,

$$\begin{aligned} D_{ab} = \frac{1}{2C}\sum _{i=1}^n \bigg [\alpha \left( 1-\mu _i(\varvec{F}^a)^{-\frac{1}{\alpha }}\right) + \alpha \left( 1-\mu _i(\varvec{F}^b)^{-\frac{1}{\alpha }}\right) \bigg ] \end{aligned}$$

Again, this reduces our reliance on the reversibility of the CTMC (though to use the SVD method discussed in the previous section, reversibility is necessary to guarantee the symmetry of *L*). Practically, particularly on smaller datasets, we have found that using

$$\begin{aligned} \bar{L}^{ab} := \frac{1}{2}\bigg [\text {diag}(\varvec{F}^a)^{\frac{1}{2}}K^{ab} \text {diag}(\varvec{F}^a)^{-\frac{3}{2}} + \text {diag}(\varvec{F}^a)^{\frac{1}{2}}K^{ab} \text {diag}(\varvec{F}^a)^{-\frac{3}{2}}\bigg ] \end{aligned}$$

and then setting $\mu _i$ to be the eigenvalues of $\bar{L}^{ab}$ can reduce the numerical instability (as a trivial example, if one of the $\varvec{F}^{\cdot }$ contains a 0, then we will not be able to calculate an *L*-matrix from that vector). On the datasets considered in this paper, both of these methods give similar results, but we present the results of this second method in our examples.

### An Alternative Evolution Equation

Our framework is flexible, and can be applied to a range of evolution equations. As an example, consider the case where

$$\begin{aligned} P^{\prime }(t) = QP(t) + B g(t) \end{aligned}$$

for some function *f*(*t*). We then know that

$$\begin{aligned} (e^{-Qt}P(t))^{\prime } = e^{-Qt}Bg(t) \end{aligned}$$

and so therefore

$$\begin{aligned} P(t) = e^{Qt}A + \int _0^{t}e^{Q(t-u)}Bg(u)du \end{aligned}$$

where *A* is some constant matrix. As $P(0) = I$, we have

$$\begin{aligned} P(t) = e^{Qt} + \int _0^{t}e^{Q(t-u)}Bg(u)du \end{aligned}$$

Now, if $\lambda$ is an eigenvalue of *Q* with eigenvector $\varvec{v}$, then

$$\begin{aligned} P(t)\varvec{v} = \bigg (e^{\lambda t} + \int _0^{t}e^{\lambda (t-u)}Bg(u)du\bigg )\varvec{v} \end{aligned}$$

Thus, $\varvec{v}$ is also an eigenvector of *P*(*t*), with eigenvalue

$$\begin{aligned} e^{\lambda t}\bigg (1 + \int _0^te^{-\lambda u}Bg(u)du\bigg ) \end{aligned}$$

which leads to

$$\begin{aligned} \text {det}(K^{ab}) = \prod _{i\in \chi } \left( e^{\lambda _i D^{ab}}\bigg (1 + \int _0^{D^{ab}}e^{-\lambda _i u }Bg(u)du\bigg )\right) \text {det}(\text {diag}(\varvec{\xi })) \end{aligned}$$

Again, this requires knowledge of the $\lambda _i$.

## Conclusions

The method introduced in this paper provides a flexible and reliable way to estimate the phylogenetic distances between a set of taxa. By combining ideas from the GTR and log-det approaches, our model has the flexibility to incorporate heterotachy across the dataset and calculate a likelihood for each set of parameters while also being practically computable. Particularly when looking at large-timescale datasets, these properties are vital in ensuring that our phylogenetic analysis is accurate, both in terms of the central estimate and the uncertainty around it.

We hope to consider extensions to this model in future work, potentially considering different distributions that could more accurately, or more flexibly, describe the variation in genetic rates. Furthermore, given the wide range of possible estimators for the stationary distribution $\varvec{\xi }$, we hope to investigate the different possibilities more systematically in order to perhaps improve the version used in this paper.

## Diagonalisability of *Q*

### Lemma 1

The matrix *Q* is diagonalisable

**Proof:** Note that, as *Q* is reversible

$$\begin{aligned} \xi _i Q_{ij} = \xi _j Q_{ji} \end{aligned}$$

Hence, this means that the matrix $R = \text {diag}(\varvec{\xi })^{\frac{1}{2}}Q \text {diag}(\varvec{\xi })^{-\frac{1}{2}}$ is symmetric as its entries are

$$\begin{aligned} R_{ij} = \xi _i^{\frac{1}{2}}Q_{ij}\xi _j^{-\frac{1}{2}} = \xi _i^{\frac{1}{2}}\bigg (\frac{\xi _j}{\xi _i}\bigg )Q_{ji}\xi _j^{-\frac{1}{2}} = \xi _i^{-\frac{1}{2}}Q_{ji}\xi _j^{\frac{1}{2}} = R_{ji} \end{aligned}$$

Thus, *R* is diagonalisable and so there exists a diagonal matrix *D* and an invertible matrix *V* such that

$$\begin{aligned} R = V^{-1}D V \end{aligned}$$

and hence

$$\begin{aligned} Q = \text {diag}(\varvec{\xi })^{-\frac{1}{2}}V^{-1}D V \text {diag}(\varvec{\xi })^{\frac{1}{2}} \end{aligned}$$

and hence, setting $W = V \text {diag}(\varvec{\xi })^{\frac{1}{2}}$, we see that

$$\begin{aligned} Q = W^{-1} D W \end{aligned}$$

as required for diagonalisability.

## Complexity

The leading-order computational complexity of our method is as follows, in terms of the number of sites *N*, the number of taxa *n*, and the number of possible site values *C*. We begin by considering the operations that only need to be carried out once.

Firstly, calculating the matrices $K^{ab}$ in (1) has complexity $\mathcal {O}(Nn^2)$, while the frequency vector calculation in (2) has complexity $\mathcal {O}(nC)$. Creating the pair-specific stationary distribution estimates in (3) has complexity of $\mathcal {O}(n^2C)$. Calculating the matrices $\bar{L}^{ab}$ in (5) has complexity $\mathcal {O}(n^2C^2)$, while computing their singular value decompositions has complexity $\mathcal {O}(n^2C^3)$. Overall therefore, our pre-computations have complexity of $\mathcal {O}(n^2N + n^2C^3)$.

We now consider the complexity of our likelihood function (7). The most complex step is computing the eigendecomposition of the $Q^{ab}$ needed to calculate (4). This is expensive, and has complexity $\mathcal {O}(n^2C^3)$, with the rest of the calculations in (7) requiring only $\mathcal {O}(n^2C^2)$ time.

However, it is the complexity of this likelihood which makes our method advantageous, as we need only sample $\mathcal {O}(C^2)$ parameters, rather than an additional $\mathcal {O}(n^2)$ inter-taxa distances (note that, while we must calculate our distance approximations from each sampled set of parameters using Eq. (6)), this simply has complexity of $\mathcal {O}(Sn^2C)$ as the singular value decompositions of the $\bar{L}^{ab}$ matrices are precomputed). Making a rough assumption that the complexity of generating *S* parameter samples is equal to the number of parameters multiplied by *S*, this means that our overall sampling complexity is $\mathcal {O}(Sn^2C^5)$, compared to $\mathcal {O}(Sn^4C^3 + Sn^2c^5)$ (note that the $\mathcal {O}(n^2N)$ may in fact still dominate, but this is common to both approaches). When fitting complex amino acid models (with $C>> 1$) on datasets with large numbers of taxa (so $n>> C$) our approach therefore provides a substantial computational saving to an extremely expensive operation.

## Metric Properties of the Log-Determinant

A metric *D* on a set *S* must satisfy three properties

$$\begin{aligned} (1) \quad&D(x,x) = 0 \quad \forall x \in S\\ (2) \quad&D(x,y) = D(y,x) \quad \forall x,y \in S\\ (3) \quad&D(x,y) + D(y,z) \ge D(x,z) \quad \forall x,y,z \in S \end{aligned}$$

The log-determinant distance $D_{ab}$ always satisfies the first two of these properties, with 1) following as, when $a=b$, we have

$$\begin{aligned} K^{ab} = \text {diag}(\varvec{f}^g) \end{aligned}$$

and so we see $D_{aa} = 0$.

The second property also follows quickly as swapping *a* and *b* simply transposes $K^{ab}$, therefore leaving its determinant unchanged.

However, for finite *N*, *D* may not satisfy 3). For example, it is undefined if the determinant of *K* is non-positive (as a trivial case, this always happens when $N < 4$ as one row will only contain 0’s). Even when restricted to positive determinants, the triangle inequality can fail - for example, with the sequences

$$\begin{aligned} g^a&= (3, 1, 0, 3, 1, 1, 3, 1, 1, 2, 1, 3, 2, 1, 3, 3, 2, 1, 1, 2)\\ g^b&= (3, 1, 3, 0, 0, 1, 3, 2, 2, 2, 2, 3, 3, 1, 2, 3, 0, 2, 1, 1)\\ g^c&= (1, 3, 0, 3, 2, 0, 1, 2, 0, 1, 2, 0, 1, 3, 2, 0, 1, 1, 3, 0) \end{aligned}$$

which gives

$$\begin{aligned} D_{ab} \approx 11 \quad D_{bc} \approx 8 \quad D_{ac} \approx 46 \end{aligned}$$

and hence

$$\begin{aligned} D_{ac} > D_{ab} + D_{bc} \end{aligned}$$

Thus, care must be taken when applying this metric to small phylogenetic datasets, although, due to the generally small state-space $\chi$ compared to large *N*, this distance will give good performance on phylogenetic datasets.

Note that in the the $N\rightarrow \infty$ limit, *D* does satisfy 3) if evolutionary distance also satisfies 3). In a general phylogenetic tree construction, this holds - given three points, *A*, *B* and *C* on a tree (representing taxa - though they need not be nodes here), their evolutionary distance is simply the distance between them on the tree, and therefore it is simple to show that *D* is a metric. Briefly, one can use the fact that there is a unique path between any pair of points. The path between *A* and *C*, $\mathcal {P}(A,C)$ can be found by choosing all points that are on exactly one of $\mathcal {P}(A,B)$ and $\mathcal {P}(B,C)$. Thus, the length of $\mathcal {P}(A,C)$ is necessarily shorter than the sum of the lengths of $\mathcal {P}(A,B)$ and $\mathcal {P}(B,C)$ as required.
